# Eating dysfunction associated with oromandibular dystonia: clinical characteristics and treatment considerations

**DOI:** 10.1186/1746-160X-2-47

**Published:** 2006-12-07

**Authors:** Spiridon Papapetropoulos, Carlos Singer

**Affiliations:** 1 Division of Movement Disorders, Department of Neurology, University of Miami, Miller School of Medicine, Miami, FL, USA

## Abstract

**Background:**

In oromandibular dystonia (OMD) abnormal repetitive contractions of masticatory, facial, and lingual muscles as well as the presence of orobuccolingual (OBL) dyskinesias may interfere with the appropriate performance of tasks such as chewing and swallowing leading to significant dysphagia and weight loss. We present here the clinical characteristics and treatment variables of a series of patients that developed an OMD-associated eating dysfunction.

**Methods:**

We present a series of patients diagnosed and followed-up at the Movement Disorders Clinic of the Department of Neurology of University of Miami, Miller School of Medicine over a 10-year period. Patients were treated with botulinum toxin injections according to standard methods.

**Results:**

Five out of 32 (15.6%) OMD patients experienced symptoms of eating dysfunction associated with OMD. Significant weight loss was reported in 3/5 patients (ranged for 13–15 lbs). Two patients regained the lost weight after treatment and one was lost to follow-up. Tetrabenazine in combination with other antidystonic medication and/or botulinum toxin injections provided substantial benefit to the patients with dysphagia caused by OMD.

**Conclusion:**

Dystonic eating dysfunction may occasionally complicate OMD leading to weight loss. Its adequate characterization at the time of history taking and clinical examination should be part of outcome measurements of the anti-dystonic treatment in clinical practice.

## Background

In oromandibular dystonia (OMD) spasms of the masticatory, facial, and lingual muscles result in repetitive and sometimes sustained jaw opening, closure, deviation, or any combination of these as well as abnormal tongue movements [1]. Although most cases are idiopathic, neuroleptic drugs can induce OMD [2,3]. OMD is very frequently associated with orobuccolingual (OBL) dyskinesias like facial grimacing, lip pursing and biting, tongue protrusion, rotation and/or dyskinesias, platysma contractions and bruxism [4]. A diagnosis of OMD carries with it a significant impact on quality of life [5]. Its treatment relies mainly on botulinum toxin type A injections that have been proven beneficial for all its subtypes [4].

Due to the anatomical distribution of the muscles affected, OMD and co-existing OBL dyskinesias are associated with disturbed perioral, oral and lingual movements that may interfere with the appropriate performance of tasks such as chewing, swallowing or talking, complicated with social embarrassment [6] and sometimes eating disorders with weight loss [7]. We present here the clinical characteristics and treatment variables of a series of patients that developed an OMD-associated eating dysfunction.

## Methods

We performed a study of consecutive OMD patients followed-up at the Movement Disorders Clinic of the Department of Neurology of University of Miami, Miller School of Medicine over a 10-year period. We identified 53 patients with primary and secondary OMD. We excluded patients who were lost to follow up after initial evaluation or had inconclusive charts (n = 21) (only patients with at least 3 evaluations were included). Five out of 32 (15.6%) OMD patients reported symptoms of dystonic eating dysfunction. All OMD patients seen in our practice are routinely asked a set of specific questions about weight loss or eating difficulties associated with OMD (have you experienced difficulties eating? Have you experienced difficulties swallowing? Have you lost weight after the onset of your disease?). All patients were examined and followed-up by the same physician (CS). Results of treatment were analyzed using a global impression scale (0 = no improvement; 1 = mild improvement; 2 = moderate improvement; 3 = marked improvement). The formulation and preparation of botulinum toxin type A (BTX-A) (BOTOX^®^, Allergan Pharmaceuticals, Irvine, CA, USA) was performed according to standard methods [8]. All patients that received BTX-A were injected bilaterally under EMG guidance, using an Allergan^® ^EMG needle. We recorded the muscles injected and the dose each muscle received. The mean dose of BTX-A for each patient was determined by adding the units injected per visit divided by the number of visits was used in the calculation of the mean dose (in units) of BTX-A in each group. The initial visit was not included since a lower than optimal dose of botulinum toxin was used.

### Case reports

#### Case 1

A 58-year-old male had been symptomatic for the preceding 3 years with a chief complaint of involuntary movements of jaw-opening triggered mainly by talking and/or eating. His symptoms made his eating difficult requiring him to bite down with effort in order to keep his mouth from opening. He wore out his regular dentures and special dentures had to be manufactured for him. Yelling would ameliorate the involuntary movements. There was no personal or family history of other neurological disorder and the patient denied any exposure to dopamine-blocking drugs. He denied any weight loss, but admitted to eating difficulty and social embracement due to his jaw-opening OMD. His neurological examination was otherwise unremarkable.

The patient was successfully treated with BTX-A injections to his lateral pterygoids (75 units/side). After 9 sessions he continues experiencing the same marked benefit and no longer complains of eating difficulties.

#### Case 2

A 48-year-old female was initially evaluated for OMD of one year evolution. She complained of intermittent involuntary movements of jaw-opening accompanied by tongue thrusting. While eating her tongue would protrude causing substantial eating and swallowing difficulties that had lead to a 15 lbs weight loss (from 110 to 95 lbs). A barium swallowing test at the time revealed her swallowing function to be moderately impaired secondary to decreased bolus preparation and decreased bolus propulsion without evidence of aspiration. After the unsuccessful injections of BTX-A (4 sessions) to her lateral pterygoids (50 u/side), she was placed on a regimen that included tetrabenazine 75 mg/day, trihexyphenidyl 3 mg/day, and lorazepam 4.5 mg/day with significant improvement of her symptoms and gradual weight gain.

#### Case 3

A 32-year-old male presented with new-onset jaw-closure spasms (jaw spasms with any kind of stimulus-able to open his mouth only 1/4 inch). This process increased in severity for three weeks, after which he could not take any food in, except through a straw. For the ensuing two months there was a gradual albeit limited improvement where he was able to open his mouth 3/4 of an inch, and from then on his condition had remained stationary. He had to change to a soft, pureed diet. Chewing would result in pain, particularly on the left mandibular area. His dystonic disorder also interfered with his speech, forcing him to keep his tongue behind the teeth to prevent from biting it. The patient was successfully treated with BTX-A injections to his masseters (50 units/side). After 4 sessions he continues to experience the same marked benefit and no longer complains of eating difficulties.

#### Case 4

A 49-year-old female presented with a 2-year history of jaw opening movements, which caused substantial drinking difficulties. A year later these movements became constant and were complicated with movements of the tongue (tongue protrusion and dyskinesias) with consequent impairment of fluid and food manipulation overlapping with chewing difficulties caused by her jaw-opening OMD. The patient reported a 15 lbs weight loss due to her condition (from 130 to 115 lbs).

She was subsequently placed on tetrabenazine (125 mg/day) with moderate benefit in the frequency and intensity of the jaw-opening movements. However, tongue protrusion and dyskinesias were not affected. The patient developed a hypokinetic extrapyramidal syndrome as a side effect to tetrabenazine therapy but insisted on continuing the drug (at a lowered dose of 75 mg/day) because of its beneficial effects on her symptoms. Within about 6 moths after the initiation of tetrabenazine the patient gained 10 lbs. In an effort to further control her symptoms the patients had trials with clonazepam and gabapentin. A combination of tetrabenazine (75 mg/day) and gabapentin (300 mg/day) improved her symptoms by a reported 75%. She also received BTX-A injections to lateral pterygoid muscles (25 units/side) without benefit. Ten years into her condition she still experiences substantial benefit from her treatment.

#### Case 5

A 56-year-old female presented with a chief complaint of involuntary jaw movements. The patient had a long (35-year) history of migraines for which she had received a number of treatments (triptans, beta-blockers and calcium-channel blockers, anti-epileptics, anti-depressants, clonazepam) with limited success. Her first trial with an atypical neuroleptic (off-label use) was 2.5 years before presentation when she had been started on ziprasidone (80 mg/day) [9]. The patient experienced a moderate decrease in the frequency and severity of her migraine attacks, but 11 months later started noticing mild involuntary movements of the tongue. Ziprasidone was gradually discontinued. Within 2 weeks jaw-opening involuntary movements were superimposed on the involuntary movements of the tongue. Gradually, her symptoms intensified, causing eating difficulties accompanied by weight loss (from 123 to 110–13 lbs). She also experienced occasional tongue and oral mucosa injuries. Her neurological examination was otherwise unremarkable. The patient had already received BTX-A injections on the lateral pterygoids at least on two occasions without success prior to visit to our clinic and declined repeat injections. She was lost to follow-up.

## Results and discussion

We present here a series of 5 patients with eating dysfunction associated with OMD. The clinical and epidemiological characteristics of our OMD cohort has been described elsewhere [10]. The prevalence of eating dysfunction in OMD in our cohort was 15.6%. The demographics, clinical characteristic and treatment details of our patients are presented in Table 1. Significant weight loss was reported in 3 out of 5 patients with eating dysfunction and OMD. Only patients with both OMD and OBL dyskinesias experienced weight loss. The weight loss ranged from 13–15 lbs (13.6% to 10.5% loss of initial body weight). Two patients regained their lost weight after treatment and one was lost to follow-up. Tetrabenazine in combination with other antidystonic medication and/or BTX-A injections provided substantial benefit to the patients with eating dysfunction caused by OMD (tetrabenazine has been particularly effective in hyperkinetic movement disorders [11]).

**Table 1:** Clinical characteristics and treatment outcomes of our patients

**Patient**	**Case 1**	**Case 2**	**Case 3**	**Case 4**	**Case 5**
Type of OMD	Jaw Opening	Jaw Opening	Jaw Closure	Jaw Opening	Jaw Opening
Etiology	Idiopathic	Idiopathic	Idiopathic	Idiopathic	Tardive

Age	58	48	32	57	50
Gender	Male	Female	Male	Female	Female
Duration	5y	1y	2y	10y	1y
Follow-up	3y	2y	1.5y	8y	1y

Other Dystonia	No	Blepharospasm	No	No	No
Orofaciolingual involvement	No	Tongue protrusion	No	Tongue dyskinesias, bruxism	Facial grimacing, tongue dyskinesia, lip biting

Botulinum toxin/sessions	Yes/9	Yes/4	Yes/4	Yes/1	Yes/2
Site(s)	Lateral pterygoids 75 u/side	Lateral pterygoids 50 u/side	Masseters 50 u/side	Lateral pterygoids 25 u/side	Lateral. pterygoids
Total	150	100	100	50	N/A
Response (GIS) to Botulinum toxin A injections	Marked	No	Moderate	No	No
Duration of response	3 months	----	3 months	-----	----
Antidystonic medications (response)	No	trihexyphenidyl lorazepam, tetrabenazine, (marked)	No	tetrabenazine, gabapentine (marked)	No

Weight loss	No	15 lbs	no	15 lbs	13 lbs
Weight gain after treatment	-----	15 lbs	-----	10 lbs	N/A

Various studies have shown that a range of swallowing difficulties accompany focal cranio-cervical dystonias such as spasmodic dysphonia and spasmodic torticollis before treatment initiation [12-14]. Cervical dystonic contractions leading to anatomical swallowing dysfunction has been proposed as a possible mechanism [15,16]. This interpretation may account for the dysphagia encountered in at least some of our OMD patients. Other proposed, previously reported mechanisms include excess duration of muscle activity, frequent co-contraction, loss of rhythmicity during chewing, and abnormalities in the chewing to swallowing transition phase [6]. These abnormalities, similar in type to those encountered in other forms of focal dystonia, may be the expression of an abnormal motor control of basal ganglia over mastication-related movement pattern generators of the brainstem [6].

Dysphagia has been also reported as a complication of BTX-A therapy in OMD [8,17,18]. However, none of our patients experienced further difficulty swallowing after receiving BTX-A injections. In fact, improvement was reported in 2 of the four of our patients who received this treatment.

Our study has some inherent limitations commonly seen in retrospective series. Documentation of all clinical and treatment variables may not be complete. However, all study patients have been evaluated and followed by the same movement disorders specialist (CS). Hence the impact of confounding factors such as inconsistencies in the diagnosis, inaccurate history, inter-examiner differences, and under-documentation were reduced. Furthermore, there are no additional evaluations of masticatory function (i.e. masticatory muscle EMG, jaw movement recording) other than weight loss (which may be influenced by many factors) and patient descriptions.

In summary, eating dysfunction was reported in 15.6% of our OMD cases. Eating dysfunction was associated with significant weight loss in three of our patients. Interestingly, only patients with OBL dyskinesias experienced weight loss. Additional difficulties included pain during eating, social embarrassment and speech disturbance. A measure of benefit was reported in the majority of our patients with BTX-A injections and oral antidystonic medications, especially tetrabenazine. Eating dysfunction associated with OMD should be identified and adequately characterized at the time of history taking and should be part of outcome measurements of the anti-dystonic treatment. Special attention has to be paid to patients with OMD and OBL dyskinesias since in our cases they experienced significant weight loss.
